# Acute effects of mango leaf extract on cognitive function in healthy adults: a randomised, double-blind, placebo-controlled crossover study

**DOI:** 10.3389/fnut.2024.1298807

**Published:** 2024-04-11

**Authors:** Fiona L. Dodd, David O. Kennedy, Jodee Johnson, Emily Haworth, Jessica P. Greener, Philippa A. Jackson

**Affiliations:** ^1^Brain, Performance, Nutrition Research Centre, Department of Psychology, Northumbria University, Newcastle upon Tyne, United Kingdom; ^2^PepsiCo, Health & Nutrition Sciences, Chicago, IL, United States; ^3^Population Health Sciences Institute, Newcastle University, Newcastle upon Tyne, United Kingdom

**Keywords:** mango leaf extract, cognition, cognitive demand, attention, stress, mood

## Abstract

**Introduction:**

Extracts made from the leaves of the edible mango plant (*Mangifera indica* L., Anacardiaceae) have a long history of medicinal usage, most likely due to the presence of high levels of mangiferin, a polyphenol compound. Previous research has demonstrated that mango leaf extract (MLE) can beneficially modulate cognitive function in both animals and humans. This study aimed to assess the effects of an acute dose of 300 mg MLE (standardised to contain ≥60% mangiferin) on cognitive performance and mood in healthy adults.

**Methods:**

In this double-blind, placebo-controlled, crossover study, 114 healthy men and women (18–43 years) received either MLE or a matched placebo at each testing visit (separated by at least 7 days). Cognitive performance (including the cognitive demand battery) and mood were measured at 30, 180, and 300 min post-dose.

**Results:**

The results showed that, compared to placebo, the group taking MLE displayed a significant increase in serial 3 s and serial 7 s subtraction errors overall. There were no other significant effects on cognitive performance.

**Discussion:**

The results of the current study suggest that the consumption of 300 mg MLE in the absence of an observed multitasking psychological stressor does not improve cognitive performance or mood at up to 300 min post-dose. Due to the very limited nature of the effects and since they were observed among many analyses, these findings should be treated with caution.

**Clinical trial registration**: http://ClinicalTrials.gov, identifier [NCT05182450].

## Introduction

Mango leaf (*Mangifera indica L*., Anacardiaceae) has been utilised as a traditional medicine for its pharmacological effects in the treatment of diseases of the lungs, gallbladder, and kidney as well as bronchitis, diabetes, malaria, cancer, and inflammation (1, 2). The bioactivity of the mango leaf extract (MLE) is believed to be due to the presence of high levels of the polyphenol, xanthone, found in the extract, specifically mangiferin (1). Xanthones are only found in a small number of plant species, like *Clusiaceae*, but are seldom ingested by humans, with the exception of mango. Antibacterial, antifungal, and anti-inflammatory properties, among others, have all been found to result from mangiferin, a naturally occurring xanthone (1). It has been speculated that, although mangiferin is structurally different from other polyphenols, its mechanism of action may be similar (3).

Previous research has demonstrated that a single dose as well as chronic administration of polyphenols can beneficially modulate cognitive function (4–6), including during cognitively demanding task performance (7). A systematic review investigating the effects of doses ranging from 10 to 200 mg/kg of mangiferin over 12–154 days on memory impairment in animal models found that all studies reported an improvement in memory, specifically spatial recognition, episodic aversive events, and short- and long-term memories (8). Furthermore, a recent study in rats demonstrated a similar effect of MLE on electrophysiological (electroencephalopathy (EEG)) spectral power as that noticed following caffeine, with a synergistic effect of co-consumption of MLE and caffeine (9). *M. indica* has also been shown to enhance brain oxygenation and physical performance (10) and improve ergogenic parameters following ischaemia-reperfusion (11) in healthy humans when consumed alongside other polyphenols. Moreover, when consumed in isolation at a dose of 500 mg, MLE modulated brain electrical activity in humans (as measured by quantitative EEG) during cognitive challenge and led to reduced ratings of fatigue at 90 min and significant percentage improvement in reaction time as compared to placebo, at 60 min post-dose (12). Taken together, these findings raise the possibility that MLE, due to its high mangiferin content, may have beneficial effects on psychological parameters.

A recent study by our group (3) expanded on the above during an assessment of the effects of a single dose of MLE (>60% of polyphenol mangiferin) on performance across a number of cognitive domains and during laboratory-induced stress in humans. The results showed that a single dose of 300 mg MLE significantly improved performance accuracy across the tasks in the battery, with domain-specific effects observed in terms of enhanced performance on a global measure of “Accuracy of Performance”, the composite measures of “Accuracy of Attention”, and “Episodic Memory” as well as improved performance across the Cognitive Demand Battery sub-section (serial 3 s, serial 7 s and Rapid Visual Information Processing (RVIP) tasks) of the assessment. These findings were observed across assessments spanning 30 min to 5 h post-dose (measured at 30, 180, and 300 min). These results provide a robust demonstration of beneficial effects following consumption of MLE, reinforcing the findings of previous research that had shown that polyphenols and polyphenol-rich extracts can improve cognitive function.

The current study sought to confirm the previous findings on cognition by replicating only the cognitive procedures of the aforementioned study (3). For this reason, the observed multitasking stressor (OMS) included in the Wightman et al. study was not included here. The aim of the present study was to further assess the effect of a single dose (300 mg) of MLE on extended cognitive task performance and mood.

## Materials and methods

### Design

The study followed a randomised, double-blind, placebo-controlled, crossover design in which the acute effects of MLE on cognitive function and mood in healthy adults were observed. The study design included two in-lab testing visits, which were conducted at least 7 days apart, following a training/familiarisation session. The study was performed in accordance with the ethical principles enshrined in the Declaration of Helsinki (1996). The trial was conducted in compliance with protocol/Good Clinical Practice (GCP)applicable regulatory requirements and commenced only when a favourable ethical opinion was obtained from Northumbria University College of Reviewers (REF: 39521). Written informed consent was obtained from all the participants. The trial is registered at ClinicalTrials.gov (NCT05182450).

### Determination of sample size

In the study by Wightman et al. (3), the required sample size for the study (*n* = 72) was calculated (GPower 3.0) on the basis of delivering adequate power (0.8) to detect a small effect size (*f* = 0.1). The study returned maximum effect sizes in the region of a medium effect size (*f* = 0.25). In the present study, a total sample size of *n* = 114 was therefore estimated to provide adequate power (0.8) to detect the small effect size apparent with respect to the primary outcome (Accuracy of Attention) at the first time point (30 min post-dose) and very good power (>0.98) to detect the larger effect sizes seen at later time points and on the primary outcome’s main effect.

### Participants

A total of 191 participants aged 18–45 years, who self-reported as being in good health, were enrolled into the study. Participants were recruited via an opportunity sample from the students and staff of Northumbria University and the general population within Newcastle upon Tyne, as well as the surrounding areas. All participants reported being free from any relevant medical condition or disease including psychiatric disorders and neurodevelopmental differences. Blood pressure and body mass index (BMI) were measured at screening and participants were enrolled into the study if blood pressure measured was <159 mmHg (systolic) or < 99 mmHg (diastolic) and the measured BMI was within the range 18.5–35 kg/m^2^. Participants confirmed that they were not currently taking any relevant pharmaceuticals (or antibiotics in the last 4 weeks) and had not taken MLE or taken part in another clinical trial within the past 30 days. A full list of inclusion and exclusion criteria can be found in Supplementary File 1. All participants provided written informed consent to participate prior to any research-related procedures being performed.

### Interventions

Participants consumed one of two interventions at each testing visit (testing visits 1 and 2): 300 mg MLE (Zynamite®—standardised to contain ≥60% mangiferin) and a matched placebo, with intervention order counterbalanced across the sample. The full composition of the active intervention capsules and placebo capsules is listed in Supplementary File 2. All investigational products were prepared according to good manufacturing practice and delivered blind from the manufacturer (Merical, CA) in individual, sealed packets identified only by a three-digit code (365 or 972). Participants were randomly allocated to a computer-generated counterbalanced randomisation schedule (www.randomization.com), which was created by the research team and which dictated the order in which the participant received the two interventions. Randomisation numbers were allocated sequentially to the participants by the researcher during the first testing visit (testing visit 1) in the order in which they arrived. Participants consumed the interventions on each visit in the laboratory under the supervision of the researcher, allowing for 100% compliance.

### Cognitive measures

#### Computerised mental performance assessment system (COMPASS)

**Figure 1:**
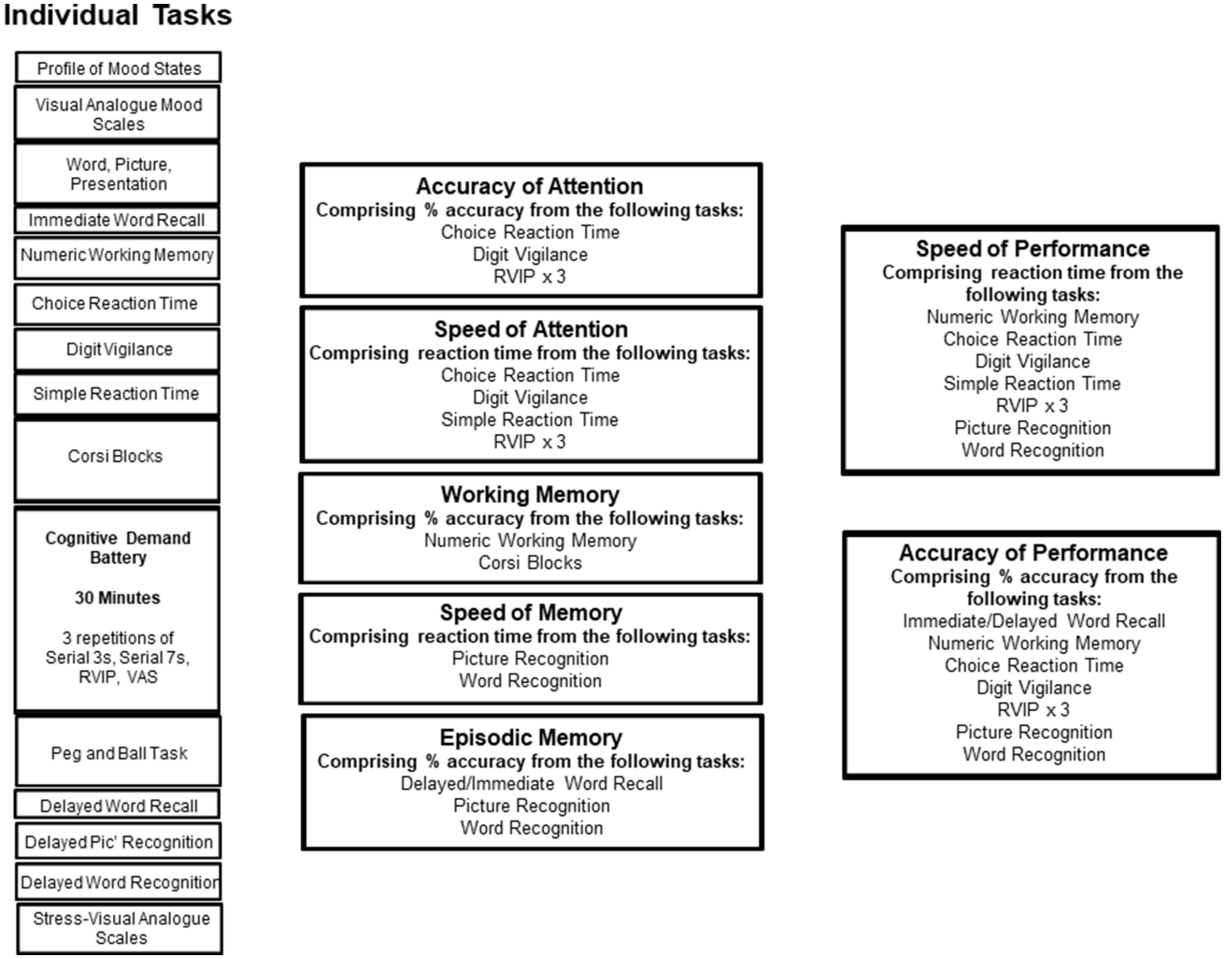
The running order of the individual tasks in the core cognitive assessment. Tasks are shown in order of completion on the left. On the right, the “cognitive domain” assessed by the tasks is shown, and the boxes to the far right show potential global measures into which data from several tasks are collapsed.

This testing system delivers a bespoke collection of tasks, with fully randomised parallel versions of each task delivered at each assessment for each individual. It has previously been shown to be sensitive to a wide range of nutritional interventions (13–18). The selection of tasks employed in the present study comprised a number of standard and ‘classic’ tasks that assess aspects of memory (working, episodic, and spatial), attention and executive function, and the ‘Cognitive Demand Battery’ (CDB), which have been shown to be sensitive in numerous studies involving nutritional interventions, including the previous study that assessed the acute cognitive effects of MLE (3). Task outcomes were collapsed into composite scores to establish if the active intervention had a global effect on a given cognitive domain that might escape significance on the component tasks. These included the names of the cognitive domains in this section should capitalised. For example; Accuracy of Attention, Speed of Attention etc. All tasks administered are described in Supplementary File 3, and the task order and the contribution of the tasks to the composite scores is shown in Figure 1.

#### Profile of mood states (POMS)

The POMS is a well-established, factor-analytically derived measure of psychological distress for which high levels of reliability and validity have been documented (19). The POMS consists of 65 adjectives rated on a 0–4 scale that can be consolidated into depression-dejection, tension-anxiety, anger-hostility, confusion-bewilderment, vigour-activity, and fatigue-inertia subscales. The latter two subscales can be interpreted as measures of fatigue and have been validated as separate factors in a number of studies. Norms have been published for a variety of patient and non-patient groups. The POMS was administered through Multi-Health Systems (MHS) Assessments Online Assessment Centre, prior to each cognitive assessment.

#### Visual analogue mood scales (VAMS)

Prior to each cognitive assessment and as part of the COMPASS battery, participants completed a series of visual analogue scales anchored by 18 antonyms relating to mood and psychological state. Participants moved a marker along the line to describe how they currently feel. Each line was scored as a percentage along the line towards the more positive antonym. The factors were labelled Alertness (11 items: alert, inattentive; lethargic, energetic; clumsy, co-ordinated; lively, sluggish; quick-witted, slow-witted; sharp, dull; exhausted, refreshed; bored, engaged; focused, unfocused; drowsy, awake; motivated, unmotivated), Stress (4 items: tense, relaxed; fearful, fearless; stressed, carefree; peaceful, troubled), and Tranquillity (3 items: tranquil, agitated; contented, discontented; friendly, hostile).

#### Stress visual analogue scales (S-VAS)

Bespoke visual analogue scales were completed on COMPASS after each battery of cognitive tasks. The scales comprised 100 mm lines (anchored as 0 = not at all and 100 = extremely) and included the following questions:

How anxious do you feel? (Not at all—Extremely).

How stressed do you feel? (Not at all—Extremely).

How relaxed do you feel? (Not at all—Extremely).

How calm do you feel? (Not at all—Extremely).

### Procedure

All data were collected at the Brain, Performance and Nutrition Research Centre (BPNRC), located on Northumbria University’s Newcastle city centre campus. Participants were required to have an initial remote screening session followed by three separate visits to the laboratory: an introductory/training/familiarisation visit and two active testing visits.

The remote screening session was completed via video/telephone call and comprised a briefing on the requirements of the study, obtaining of informed consent via completion of an online consent form, medical history, collection of demographic data, and completion of the Caffeine Consumption Questionnaire (CCQ).

The introductory/training visit to the laboratory began with in-person consent and confirmation of eligibility. Physical eligibility measures of height, weight, blood pressure (BP), and waist-to-hip ratio (WHR) were obtained. Participants were then given the opportunity to ask questions about the study, familiarise themselves with the laboratory environment, and train on the cognitive/mood tasks they would complete at testing. An abbreviated version of the Pittsburgh Sleep Questionnaire (20) was also administered at this session in order to record the average number of hours of sleep per night. This information was then used to ensure the participants had experienced a typical night’s sleep prior to attending each testing visit.

The methodology at both testing visits was identical, with the exception of the intervention the participant consumed. Upon arrival, participants were asked to confirm that they had followed the pre-testing instructions, specifically (i) to eat a standardised breakfast of cereal and/or toast at home no later than 1 h prior to arrival at the lab, (ii) to avoid caffeine for 12 h prior to attending the lab, (iii) to avoid vigorous physical activity for 24 h prior to testing, (iv) to have a typical night’s sleep prior to each visit, and (v) to avoid alcohol 24 h prior to testing. Participants were advised they would be required to arrive in the same state for the second testing visit as their first testing visit, or they would need to reschedule. This included eating the same breakfast, having a typical night’s sleep and avoiding alcohol, strenuous physical exercise, and caffeine for the requisite amount of time prior to their attendance. Testing was conducted in a suite of dedicated temperature-controlled university laboratories with participants visually isolated from each other.

**Figure 2:**
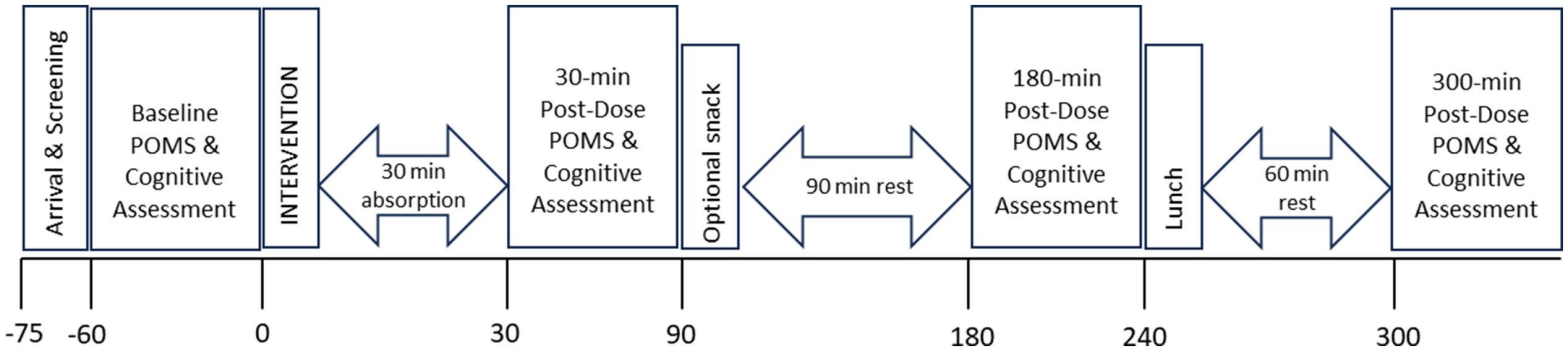
The timeline of the testing day for an individual participant, showing the cognitive assessment schedule.

Upon arrival at each testing visit, the participants were screened for continued eligibility and then completed the POMS followed by a 60-min computerised cognitive/mood assessment (COMPASS—including the VAMS, individual cognitive tasks, CDB, and S-VAS). After the first set of cognitive/mood assessments, the participants took their intervention capsule with water under the supervision of a member of the research team. As this was a replication of Wightman et al. study and because they observed significant effects of the intervention within 30 min of administration, the same timeframe for absorption was applied here. Participants then underwent cognitive/mood assessments identical to the above at 30, 180, and 300 min post-dose. Following the completion of the 30-min post-dose assessment, the participants were given the option of a snack a decaffeinated hot tea or coffee and plain digestive biscuits (replicated at testing visit 2). Following completion of the 180-min post-dose assessment, the participants were provided with a standardised lunch comprising of a cheese sandwich on white bread with butter, crisps, and a custard pot (replicated at testing visit 2). No alternative snacks or lunches were offered. Upon completion of testing visit 2, participants completed a “treatment guess” form and were fully debriefed, thanked, and compensated for their time. See Figure 2 for a schematic depicting the procedure during testing visits 1 and 2.

### Statistical analysis

All outcomes were analysed using SPSS (version 28.0, IBM corp.). Multilevel modelling was used to fit the composite or individual score for each outcome measure. Intervention (MLE, Placebo) and Time (30, 180, 300 min post-dose) were entered into the model as fixed effects along with their interaction and respective baseline score as a covariate. Repetition (1-3) was included as an additional fixed effect for the analysis of the CDB outcome measures. Participant was entered as a random effect where appropriate, as determined by Schwartz’s Bayesian Criteria. The covariance structures of the residuals were modelled as appropriate. Significant interaction effects were analysed further using pairwise comparisons and adjusted for multiple comparisons according to Sidak.

## Results

**Figure 3:**
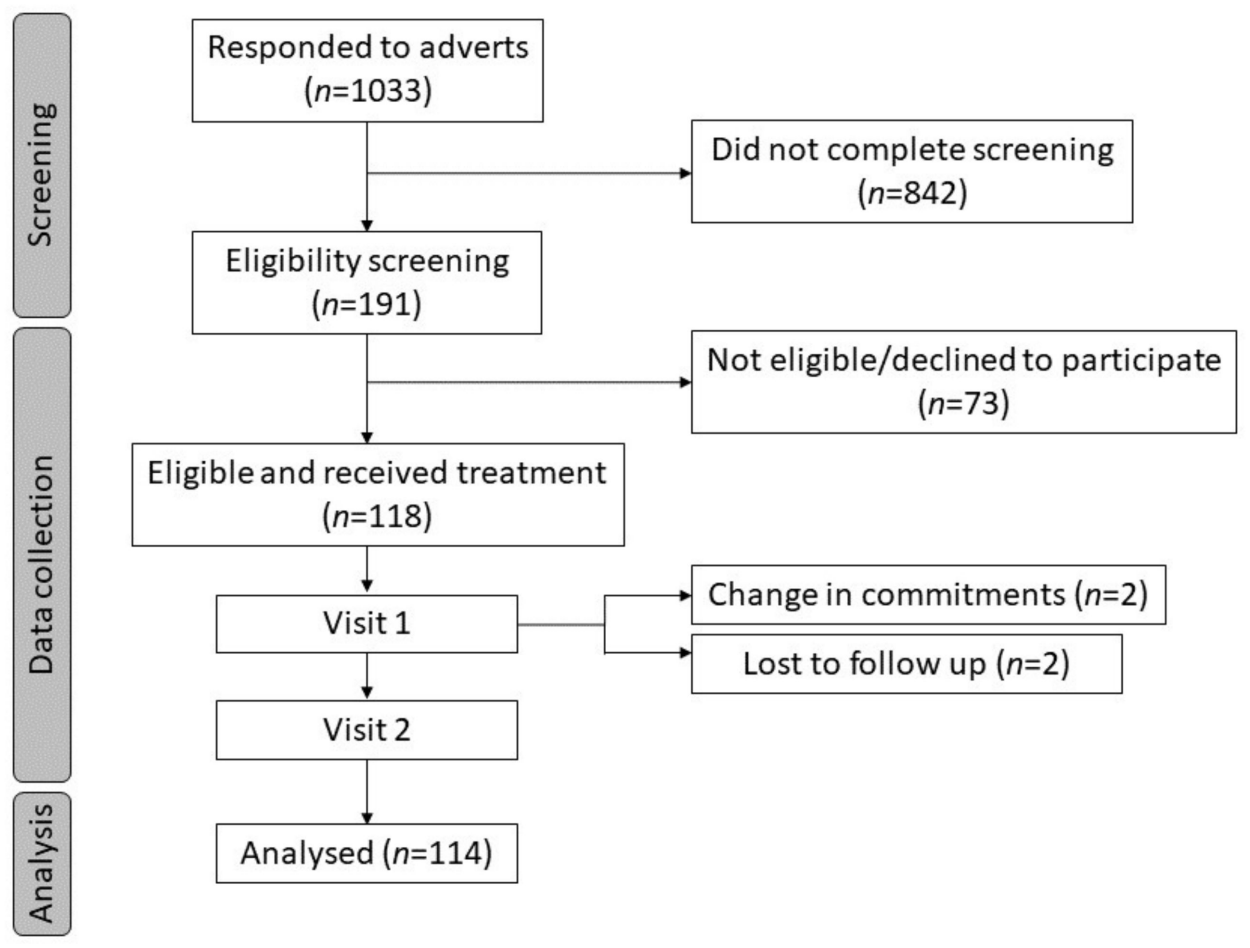
Participant disposition flowchart.

A total of 118 participants were randomised to the interventions. Four participants withdrew post-randomisation following testing visit 1, leaving a total of 114 complete datasets that were included in the analysis. See Figure 3. Missing data were presumed to be missing at random and were estimated within the analysis using the restricted maximum likelihood (REML) method.

### Demographic and baseline characteristics

Participant demographics and baseline characteristics are summarised in Tables 1, 2.

**Table 1:** Participant characteristics at enrolment for continuous variables (*N* = 114).

Measure	Mean	SD
Age (years)	25.37	6.40
Education (years)	16.35	2.76
Normal hours sleep per night	7.62	1.18
Fruit and vegetable consumption (portions/day)	3.20	1.74
Caffeine consumption (mg/day)	80.41	79.10
Systolic BP (mm/Hg)	120.67	13.25
Diastolic BP (mm/Hg)	77.10	8.39
Heart rate (beats per minute)	77.50	11.70
BMI (kg/m^2^)	24.40	3.54

**Table 2:** Participant characteristics at enrolment for categorical variables (*N* = 114).

Measure	Count	%
Men/women	47/67	41/59
Race		
White	71	62.3
Black	9	7.9
Asian	22	19.3
Chinese	3	2.6
Other	9	7.9
Wear glasses (yes/no)	27/87	24/76
Handedness (left/right)	10/104	9/91
Dietary habits		
Omnivore	100	87.7
Vegetarian	10	8.8
Pescatarian	1	0.9
Vegan	2	1.8
Other	1	0.9
Highest level of education		
Entry level (entry level certificates)	0	0.0
Level 1 (NVQ level 1, GCSE D-G, BTEC Intro)	4	3.5
Level 2 (NVQ level 2, GCSE A*-C, Young Apprenticeships, BTEC 1 or 2)	49	43
Level 3 (NVQ level 3, AS & A-levels, BTEC 3)	2	1.8
Level 5 (NVQ level 5, HND, HNC, Dip HE/FE, Found. Deg, BTEC 5)	4	3.5
Level 6 (Bachelors Degree, GradDip, Grad Cert., BTEC 6)	35	30.7
Level 7 (Masters, PGDip, PG Cert, BTEC 7)	17	14.9
Level 8 (Doctorates, BTEC 8)	3	2.6

### Compliance and intervention blinding

As participants consumed the interventions on each visit in the laboratory under the supervision of the researcher, compliance was at 100%. The success of blinding was confirmed via “treatment guess” at the end of the study (final testing visit). The chi-squared analysis confirmed that participants were not able to correctly identify whether they had received MLE or placebo [X^2^ (1, *N* = 114) = 0.04, *p* = 0.851] at their final visit. Specifically, 53% of participants who received placebo and 54% of participants who had received MLE at their final visit guessed that they had been given placebo.

### COMPASS task battery

No effect of the intervention was observed for the primary outcome measure Accuracy of Attention, nor any other of the composite or individual task scores.

### Cognitive demand battery

**Figure 4:**
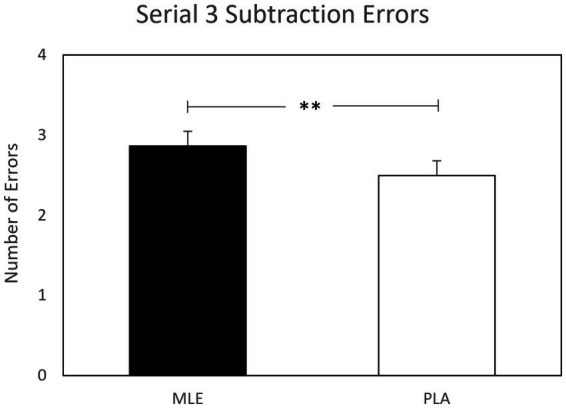
Effect of interventions on number of serial 3 subtractions errors during the cognitive demand battery. Data presented are estimated marginal means (±SE) derived from the linear mixed model for serial 3 subtraction errors. **p < 0.01. MLE, Mango leaf extract; PLA, Placebo.

**Figure 5:**
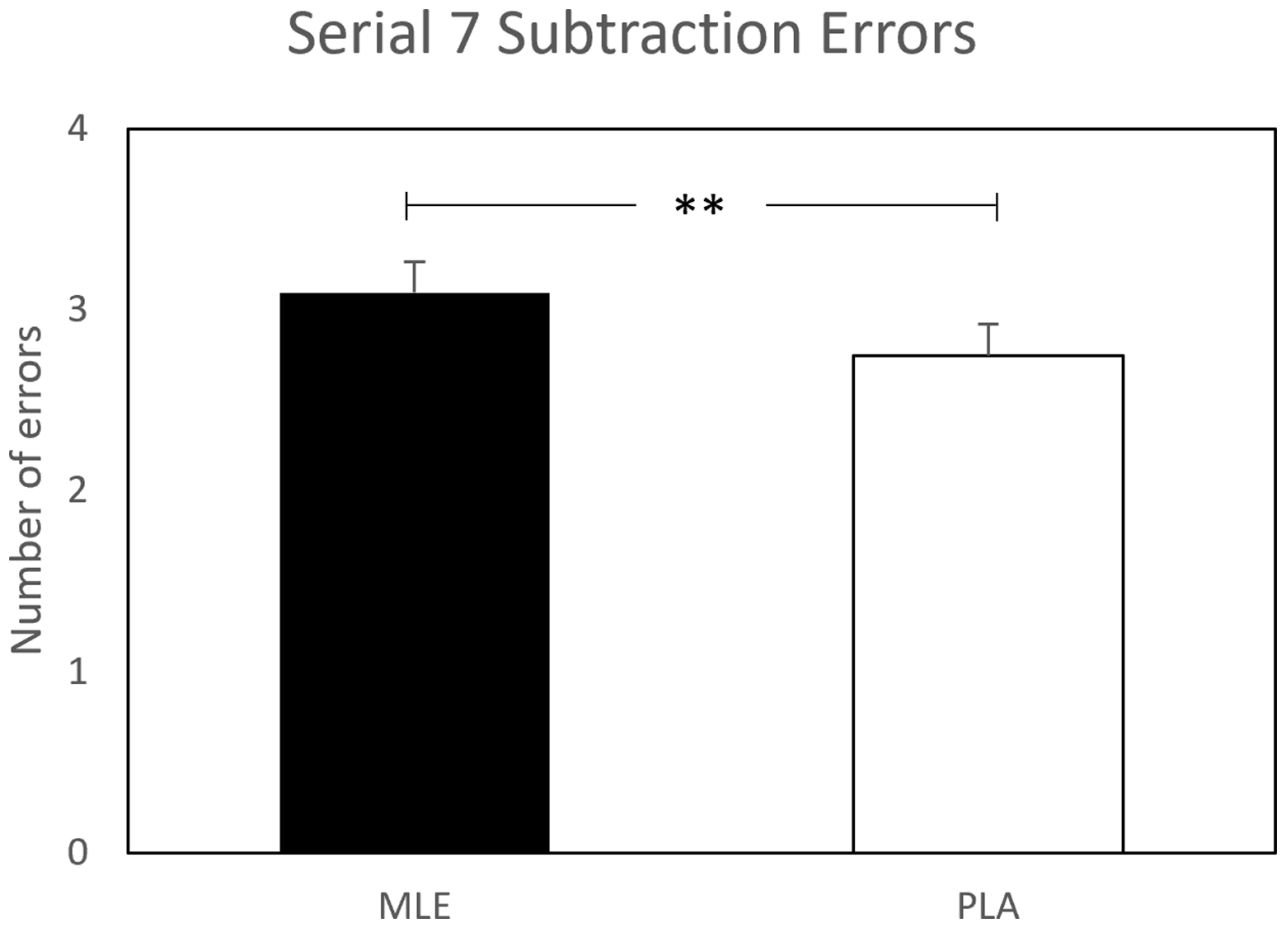
Effect of interventions on number of serial 7 subtractions errors during the cognitive demand battery. Data presented are estimated marginal means (±SE) derived from the linear mixed model for serial 7 subtraction errors. **p < 0.01. MLE, Mango leaf extract; PLA, Placebo.

A main effect of intervention was observed for serial subtractions errors for both serial 3 s and serial 7 s subtractions. Investigation revealed that there were significantly fewer errors following placebo (*M* = 2.50; SEM = 0.18) as compared to MLE (*M* = 2.86; SEM = 0.18), for serials 3 s errors [*F* (1, 561.94) = 7.77, *p* < 0.01], and significantly fewer errors following placebo (*M* = 2.75; SEM = 0.19) as compared to MLE (*M* = 3.10; SEM = 0.19), for serial 7 s errors [*F* (1, 475.73) = 7.37, *p* < 0.01]. See Figures 4, 5. No other cognitive performance effects were observed.

### Mood measures

No effect of intervention was observed for any of the mood outcome measures.

Tables of all outcome measures can be found in Supplementary File 4.

## Discussion

The results of the current study suggest that the consumption of 300 mg MLE does not improve cognitive performance or mood, at up to 300-min post-dose. The limited effects that were observed followed a pattern of worsening performance, with main effects across both serial 3 s and serial 7 s subtraction errors revealing that, compared to placebo, MLE led to an increase in subtraction errors overall.

The negative effects observed here for the consumption of 300 mg MLE are somewhat unexpected, particularly since previous research of this extract has demonstrated predominantly positive effects across physiological, cognitive, and mood parameters in humans and animals (3, 8, 9, 12). As a consequence and, given the small number of negative effects among many analyses, these results should be treated with caution.

The current study intended to confirm previous cognitive research on this mango extract and was a replication of the study by Wightman et al. (3), who administered the same extract and implemented similar study procedures to those described here. Their study demonstrated that 300 mg MLE led to improved Accuracy of Performance, Accuracy of Attention, Episodic Memory, and, specifically in relation to the findings here, improved performance on serial 3 s and serial 7 s subtractions tasks (demonstrated by an increase in the number of correct responses). The absence of extract-related cognitive effects in the present study in light of the series of positive effects observed previously is difficult to explain and may be as a result of inexorable methodological differences, specifically the sample of participants studied. It is pertinent to mention, however, that the participant criteria for inclusion and exclusion in the present study was matched to that of the Wightman study. Differences in batches may also provide some explanation for the disparate findings in this study. Here, MLE was standardised to contain ≥60% mangiferin, and variations in the composition of the extract as a consequence of the percentage of mangiferin (however small) have the potential to impact the results. However, previous research has demonstrated positive effects of this polyphenol, irrespective of differences in batch or composition of the extract investigated (3, 8, 12).

Of note here is a key methodological difference between the two studies; one possibility is that the observed multitasking stressor (OMS) included in the Wightman study (but not included here) led to an unexpected influence with regards to the impact of the extract on cognitive performance. Indeed, simple comparisons of the mood/stress levels observed here with the aforementioned study suggest a more pronounced effect upon these measures in the Wightman study (even when taking differences in the sample into consideration). In the study by Wightman et al., a 5-min OMS assessment was administered at baseline and again following the 30-min post-dose cognitive assessment. It has been demonstrated previously that the OMS procedure modulates subjective and physiological measures of stress for an extended period—during, after, and in anticipation of its administration. For example, cortisol levels have been shown to be elevated at 15 min before the OMS and staying elevated until their decline approximately 60 min post-OMS (21). Mangiferin is known to possess anti-inflammatory, immunomodulatory, antioxidant, and neuroprotective properties (22–26) and has been shown to alleviate corticosterone (27) and lipopolysaccharide-induced anxiety in mice (28), ameliorating neurobehavioural deficits (27). Taking this into consideration, it is possible that the additional demand placed on cognitive resources as a consequence of the OMS gave rise to the cognitive improvements observed following MLE previously and may provide some explanation as to why similar cognitive effects were not observed in the present study. It is important to note, however, that beneficial cognitive effects of MLE have been observed in healthy animal models when the demand experienced has been arguably lower than that experienced here (29).

There were, however, methodological limitations of the present research. Participants were not required to follow a low polyphenol diet in the days prior to their attendance at each of the study visits (as per Wightman et al., whose study this research sought to replicate). Since mangiferin (the compound considered to be active within MLE) is itself a polyphenol, variability in its levels at baseline has the potential to impact the results observed. Future research involving this extract would benefit from participants being advised to follow a low polyphenol diet 48 h prior to testing to control for this outcome. We also acknowledge that differences in food intake on testing days between participants may have influenced study findings; for example, breakfast items chosen on the morning of testing, as well as the optional snack chosen during the testing day. However, individuals were required to have the same food at testing visit 2 as they had at testing visit 1, so the food consumed at breakfast, during the optional snack, and at lunch remained consistent within the participants. Consequently, the impact is likely to be minimal.

Due to the sparse research into MLE and its cognitive effects in human participants, in addition to the disparate findings here with that of Wightman et al. (3), future studies would benefit from exploring this extract further with respect to cognition and mood. An investigation of the dose–response would be of interest since positive psychological effects of mangiferin have been observed in animals at doses as low 10 mg/kg and as high as 200 mg/kg. A similar exploration of the dose–response relationship in humans is warranted, since, at present, research is limited to the observation of positive cognitive and mood effects at doses of 300 and 500 mg (3, 12). Furthermore, an exploration of the chronic effects of MLE would be beneficial in order to determine if longer-term supplementation leads to a cumulative effect with regards to cognition.

Overall, the findings of the present study suggest that consumption of 300 mg MLE, in the absence of an observed multitasking psychological stressor, does not improve cognitive performance or mood, at 30, 180, or 300 min post-dose. The specific effects that were observed as a result of MLE followed a pattern of worsening performance. However, due to the very limited nature of the effects and since they were observed among many analyses, these findings should be treated with caution.

## Data availability statement

The raw data supporting the conclusions of this article will be made available by the authors, without undue reservation.

## Ethics statement

The studies involving humans were approved by the Northumbria University Ethical Approval System (ref: 39521). The studies were conducted in accordance with the local legislation and institutional requirements. The participants provided their written informed consent to participate in this study.

## Author contributions

FD: Writing – original draft, Writing – review & editing. DK: Writing – review & editing. JJ: Writing – review & editing. EH: Writing – review & editing, Writing – original draft. JG: Writing – original draft, Writing – review & editing. PJ: Writing – review & editing.
