# Supplementary material for: Acute effects of mango leaf extract on cognitive function in healthy adults: a randomised, double-blind, placebo-controlled crossover study
Source: Front Nutr. 2024 Apr 11;11:1298807. doi: 10.3389/fnut.2024.1298807 (PMC11043474; doi:10.3389/fnut.2024.1298807)
Supplement: Supplementary file 1 [file Data_Sheet_1.zip › Supplementary File 1.docx]

**Supplemental File 1 - Full list of all inclusion/exclusion criteria**

**Inclusion Criteria**

- Participants are aged 18 to 45 years, inclusive
- Participants self-report that they are in good health
- Willingness to abstain from consumption of caffeine within 12 h of testing
- Willing to abstain from alcohol consumption and avoid vigorous physical activity for 24 h prior to all test visits
- Willing to refrain from ‘over the counter’ medications (e.g. pain medication) and stimulant medication for 12 hours, seasonal allergy/hayfever nasal antihistamine medications for 24 hours and oral antihistamines for 48 hours prior to all test visits
- Understanding the study procedures and willing to provide informed consent to participate in the study and authorization to release relevant protected health information to the study investigator

##

## Exclusion criteria

Participants complying with at least one of the following criteria were not eligible:

- Failure to meet any one of the inclusion criteria
- Have any pre-existing medical condition/illness which will impact taking part in the study. NOTE: the explicit exceptions to this are controlled hay fever. There may be other, unforeseen, exceptions and these will be considered on a case-by-case basis; i.e. participants may be allowed to progress to screening if they have a condition/illness which would not interact with the active intervention or impede performance.
- Current use of prescription medication (no antibiotics within the last 4 weeks) NOTE: the explicit exceptions to this are contraceptive treatments for female participants, and those taken ‘as needed’ in the treatment of asthma and hay fever. As above, there may be other instances of medication use which, where no interaction with the active intervention is likely, and which would not be expected to have any impact on brain function, participants may be able to progress to screening
- Have sleep disorders or are taking sleep aid medication
- Major trauma or major surgical event within 6 months of screening
- Extreme dietary habits, as judged by the Investigator (high fat, very high protein diets, intermittent fasting, etc.)
- Exposure to MLE within 30 d prior to screening
- History of cancer in the prior two years, except for non-melanoma skin cancer
- Have a visual impairment that cannot be corrected with glasses or contact lenses (including colour blindness)
- Food allergies/intolerances/sensitivities to any ingredients in the study products and study meals (including related foods/beverages/products) and or unwillingness to eat or dislike for the study meals.
- Self-report excessive leisure time physical activity (> 7 strenuous bouts per week)
- Have a current or chronic gastrointestinal, sleep, or psychiatric disorders including medically diagnosed anxiety and depression
- Work night shifts or follow a variable work pattern that results in irregular sleep pattern
- Are pregnant, trying to get pregnant or lactating
- Smoke tobacco, vape nicotine or use nicotine replacement products (including occasional social smoking)
- Illegal/recreational drug use
- Fail to demonstrate adequate minimal performance on lab, computer-based cognitive tasks
- Have participated in another clinical trial within past 30 days
- Have high blood pressure (systolic over 159 mm Hg or diastolic over 99 mm Hg)
- Have a Body Mass Index (BMI) outside of the range 18.5-35 kg/m2
- Have a diagnosed neurological condition, or learning/behavioural or neurodevelopmental differences (e.g. dyslexia, autism, ADHD).
- Excessive caffeine intake (>500 mg per day)
- Have taken dietary supplements e.g. Vitamins, omega 3 fish oils etc. in the last 4 weeks (Note: participation is possible following a 4-week supplement washout prior to participating and for the duration of the study on the proviso that the supplements they are taking are out of choice and not medically prescribed or advised)
- Have any health condition that would prevent fulfilment of the study requirements (this includes non-diagnosed conditions for which no medication may be taken)
- Has been diagnosed with/ undergoing treatment for alcohol or drug abuse in the last 12 months
- Have been diagnosed with/ undergoing treatment for a psychiatric disorder in the last 12 months
- Suffers from frequent migraines that require medication (more than or equal to 1 per month)
- Any known active infections
- Are non-compliant with regards consumption of the intervention
- Does not have a bank account (required for payment)
