# Supplementary material for: Acute effects of mango leaf extract on cognitive function in healthy adults: a randomised, double-blind, placebo-controlled crossover study
Source: Front Nutr. 2024 Apr 11;11:1298807. doi: 10.3389/fnut.2024.1298807 (PMC11043474; doi:10.3389/fnut.2024.1298807)
Supplement: Supplementary file 1 [file Data_Sheet_1.zip › Supplementary File 2.docx]

**Supplemental file 2 – Composition of active intervention (Zynamite® – standardized to contain ≥ 60% mangiferin) and placebo**

Table 1. Formulation of active intervention (Zynamite®) and placebo capsules

| **Active Intervention** | **Placebo** |
| --- | --- |
| Size 00 yellow bovine gelatin capsule^1^ | Size 00 yellow bovine gelatin capsule^1^ |
| 300 mg mango leaf extract^2^ | - |
| 185.625 mg microcrystalline cellulose | 388.500 mg microcrystalline cellulose |
| 5.000 mg silicon dioxide | 4.000 mg silicon dioxide |
| 9.375 mg magnesium stearate | 7.500 mg magnesium stearate |

^1^ Capsules will be yellow to help disguise potential differences in colour/particle size of powders

^2^ Mango leaf extract (Zynamite® – standardized to contain ≥ 60% mangiferin) is supplied by PLT Health Solutions, Inc
