# Supplementary material for: Acute effects of mango leaf extract on cognitive function in healthy adults: a randomised, double-blind, placebo-controlled crossover study
Source: Front Nutr. 2024 Apr 11;11:1298807. doi: 10.3389/fnut.2024.1298807 (PMC11043474; doi:10.3389/fnut.2024.1298807)
Supplement: Supplementary file 1 [file Data_Sheet_1.zip › Supplementary File 3.docx]

**Supplemental file 3 – Description of tasks included in Computerised Mental Performance Assessment System (COMPASS)**

Cognitive function will be assessed using the Computerised Mental Performance Assessment System (COMPASS). This testing system delivers a bespoke collection of tasks, with fully randomised parallel versions of each task delivered at each assessment for each individual. The battery has been in use within our own laboratory for over 10 years and is now commercially available for other research organisations and is currently in use within a number of UK, New Zealand, and Australian Universities and research organisations. The selection of tasks employed here comprised a number of standard and ‘classic’ tasks that assess aspects of memory (working, episodic, spatial), attention and executive function. One potential advantage of the COMPASS battery is the possibility of collapsing the task outcomes into composite scores which can be useful to establish if the active intervention has a global effect on a given cognitive domain that might escape significance on the component tasks. The composite scores were calculated by calculating the average of the reaction times (msecs) or % accuracy for all of the tasks that contribute (as shown in Figure 1 in the main paper). Two additional global scores were also calculated using Z scores – Speed of Performance – comprising reaction time (msec) data from all tasks that return this data (with the exception of Peg and Ball); and Accuracy of Performance – comprising % accuracy data from all of the tasks that return this data.

*Episodic long-term Memory Tasks:*

**Picture presentation:** Fifteen colour photographic images of objects were presented sequentially on screen for the participant to remember at the rate of 1 every 3 s, with a stimulus duration of one second.

**Word Presentation:** A unique set of fifteen words were presented. Words were selected at random from a large bank of words (MRC Psycholinguistic Database) matched for word length, frequency, familiarity and concreteness. Stimulus duration was one second, as was inter-stimulus duration.

**Immediate and Delayed Word Recall:** Participants wrote down as many of the 15 words that they were presented during the stimulus presentation period immediately after this period and also during the delayed recall/recognition period. This task was scored for accuracy only.

**Delayed Picture Recognition:** A series of 30 pictures were displayed on the screen, one at a time (all target pictures shown during Picture Presentation plus an equal number of decoys). For each stimulus participants selected ‘Yes’ or ‘No’ to indicate if they had seen the picture before or not. The task outcomes included accuracy and reaction time.

**Delayed Word Recognition:** A series of 30 words (all target words shown during Word Presentation plus an equal number of decoys) were displayed on the screen one at a time. For each stimulus participants selected ‘Yes’ or ‘No’ to indicate if they had seen the word before or not. The task outcomes included accuracy and reaction time.

*Attention:*

**Digit Vigilance Task:** A target digit was randomly selected and constantly displayed to the right of the computer screen. A series of digits were then presented in the centre of the screen at the rate of 80 per minute and the participant was required to press the YES button as quickly as possible every time the digit in the series matched the target digit. The task lasted 2 min and there were 30 stimulus–target matches. Task outcomes were accuracy, mean reaction time and number of false alarms.

**Choice Reaction Time:** An arrow appeared on the screen pointing to the left or to the right. Participants responded with a left or right key press corresponding to the direction of the arrow. There was a randomly varying inter-stimulus interval of between 1 and 3 seconds for a total of fifty stimuli. The task outcomes were accuracy and mean reaction time for correct responses.

**Simple Reaction Time:** Participants responded with a single key press every time an upwards pointing arrow appeared on the screen. There was a randomly varying inter-stimulus interval of between 1 and 3 seconds for a total of fifty stimuli. Task outcomes were accuracy and mean reaction time.

*Working Memory:*

**Sternberg Numeric Working Memory task:** A series of five single digits were displayed on the screen, one at a time. Participants were required to try to memorise these numbers as they appeared. Once the series was complete, 30 single digits were displayed one at a time and the participants responded to indicate if each number was presented in the previous list or not. The task was repeated three times with different digits. The task outcomes included accuracy and reaction time.

**Corsi Blocks:** Spatial Working Memory Task. Nine blue squares on a black background were displayed on the screen. Some of the blue squares changed to red and back to blue again in a sequence. Participants were required to remember this sequence. The task was repeated five times at each level of difficulty with the sequence span increasing from 4 upwards, until the participant could no longer correctly recall the sequences. The task outcome was ‘span score’, and this was calculated as the average of the last 3 correctly completed trials. For example, if the participant correctly responded to all five Level 4 trials and only one Level 5 trial, their span score would be 4.3 [(4 + 4 + 5)/3].

*Executive Function*

**Peg and Ball:** Two configurations were shown on the screen. In each there were three coloured balls (blue, green, red) on one of 3 pegs. The configuration at the top of the screen was the goal configuration and participants arranged the balls on the starting configuration (shown in the centre of the screen) to match the position of balls in the goal configuration. They were required to do this in the least number of moves possible. Task outcomes included average thinking time, completion time and errors.

**COMPASS Cognitive Demand Battery**

The objective of this battery was to assess the impact of the active intervention on speed/accuracy and mental fatigue during continuous performance of cognitively demanding tasks.

Participants completed the 10-minute battery of tasks three times in immediate succession (i.e. for a continuous period of 30 minutes) as part of each cognitive assessment.

Application of this battery has been shown to reliably increase self-ratings of ‘mental fatigue’ and to be sensitive to a number of herbal and natural interventions (1-5). Particularly relevant here, the battery has been shown to be sensitive to performance enhancement by single doses of cocoa-flavanols (6), caffeinated products (1, 4) and mango leaf extract (7).

*The 10-minute battery comprises:*

**Serial 3s subtraction task** (2 mins): Computerised versions of the serial subtraction tasks were implemented using tests of 2-minute duration. Participants were required to count backwards in threes from a given number as quickly and as accurately as possible using the number keys to enter each response. A random starting number between 800 and 999 was presented on the computer screen, which was cleared by the entry of the first response. The task was scored for number of correct responses and number of errors. In the case of incorrect responses, subsequent responses were scored as positive if they were scored as correct in relation to the new number.

**Serial 7s subtraction task** (2 mins): This was identical to the serial threes task with the exception that it involved the serial subtraction of sevens.

**Rapid Visual Information Processing task** (RVIP – 5 mins): The participant was required to monitor a continuous series of digits for targets of three consecutive odd or three consecutive even digits. The digits are presented at the rate of 100 per minute and the participant responded to the detection of a target string by pressing the response button as quickly as possible. The task was continuous and lasted for 5 minutes, with 8 correct target strings being presented in each minute. The task was scored for percentage of target strings correctly detected, average reaction time for correct detections, and number of false alarms.

**‘Mental fatigue’ visual analogue scale**; Participants rated their current subjective ‘mental fatigue’ state by making a mark on a 100 mm line with the end points labelled “not at all” (left hand end) and “very much so” (right hand end).

1. Kennedy DO, Scholey AB. A glucose-caffeine 'energy drink' ameliorates subjective and performance deficits during prolonged cognitive demand. Appetite. 2004;42(3):331-3.

2. Reay JL, Kennedy DO, Scholey AB. Single doses of Panax ginseng (G115) reduce blood glucose levels and improve cognitive performance during sustained mental activity. J Psychopharmacol. 2005;19(4):357-65.

3. Reay JL, Kennedy DO, Scholey AB. Effects of Panax ginseng, consumed with and without glucose, on blood glucose levels and cognitive performance during sustained 'mentally demanding' tasks. J Psychopharmacol. 2006;20(6):771-81.

4. Kennedy DO, Haskell CF, Robertson B, Reay J, Brewster-Maund C, Luedemann J, et al. Improved cognitive performance and mental fatigue following a multi-vitamin and mineral supplement with added guaraná (Paullinia cupana). Appetite. 2008;50(2-3):506-13.

5. Kennedy D, Okello E, Chazot P, Howes MJ, Ohiomokhare S, Jackson P, et al. Volatile Terpenes and Brain Function: Investigation of the Cognitive and Mood Effects of Mentha × Piperita L. Essential Oil with In Vitro Properties Relevant to Central Nervous System Function. Nutrients. 2018;10(8).

6. Scholey AB, French SJ, Morris PJ, Kennedy DO, Milne AL, Haskell CF. Consumption of cocoa flavanols results in acute improvements in mood and cognitive performance during sustained mental effort. J Psychopharmacol. 2010;24(10):1505-14.

7. Wightman EL, Jackson PA, Forster J, Khan J, Wiebe JC, Gericke N, et al. Acute Effects of a Polyphenol-Rich Leaf Extract of Mangifera indica L. (Zynamite) on Cognitive Function in Healthy Adults: A Double-Blind, Placebo-Controlled Crossover Study. Nutrients. 2020;12(8).
