# Supplementary material for: Acute effects of mango leaf extract on cognitive function in healthy adults: a randomised, double-blind, placebo-controlled crossover study
Source: Front Nutr. 2024 Apr 11;11:1298807. doi: 10.3389/fnut.2024.1298807 (PMC11043474; doi:10.3389/fnut.2024.1298807)
Supplement: Supplementary file 1 [file Data_Sheet_1.zip › Supplementary File 4.docx]

# Supplemental file 4 – Data Tables

# Composite Scores

**Table 1**. Performance on the cognitive composite scores. The Baseline data are raw means (plus SEM) collected prior to dosing. Data presented from the 30-, 180-, 300-minute post dose (p.d.) assessments are estimated means (plus SEM), derived from the linear mixed model analysis, with the final two columns showing test statistics (F) and associated probabilities (p) for each of the fixed effects included in the model.

|  |  |  | Baseline | |  | 30 min p.d. | |  | 180 min p.d. | |  | 300 min p.d. | |  |  |  |
| --- | --- | --- | --- | --- | --- | --- | --- | --- | --- | --- | --- | --- | --- | --- | --- | --- |
|  |  | N | Mean | SEM | N | Mean | SEM | N | Mean | SEM | N | Mean | SEM |  | F | p |
| Speed of Performance | MLE | 111 | -0.06 | 0.06 | 113 | 0.08 | 0.04 | 112 | -0.02 | 0.04 | 112 | -0.03 | 0.04 | Intervention | 0.08 | 0.785 |
|  | PLA | 113 | -0.02 | 0.06 | 113 | 0.04 | 0.04 | 113 | 0.01 | 0.04 | 111 | -0.04 | 0.04 | Inter*Time | 0.61 | 0.547 |
| Accuracy of Performance | MLE | 111 | 0.24 | 0.06 | 113 | -0.03 | 0.04 | 112 | -0.07 | 0.04 | 112 | -0.16 | 0.04 | Intervention | 0.93 | 0.338 |
|  | PLA | 113 | 0.23 | 0.05 | 113 | 0.03 | 0.04 | 113 | -0.06 | 0.04 | 111 | -0.14 | 0.04 | Inter*Time | 0.45 | 0.637 |
| Speed of Attention | MLE | 112 | -0.16 | 0.06 | 113 | 0.11 | 0.05 | 112 | -0.03 | 0.05 | 112 | 0.02 | 0.05 | Intervention | 0.03 | 0.854 |
|  | PLA | 113 | -0.12 | 0.06 | 113 | 0.07 | 0.05 | 113 | 0.06 | 0.05 | 111 | -0.01 | 0.05 | Inter*Time | 1.73 | 0.179 |
| Accuracy of Attention | MLE | 112 | 0.24 | 0.09 | 113 | -0.03 | 0.08 | 112 | -0.04 | 0.08 | 112 | -0.24 | 0.08 | Intervention | 0.31 | 0.579 |
|  | PLA | 113 | 0.27 | 0.08 | 113 | -0.02 | 0.08 | 113 | -0.03 | 0.08 | 111 | -0.17 | 0.08 | Inter*Time | 0.18 | 0.834 |
| Speed of Memory | MLE | 112 | 0.09 | 0.08 | 114 | 0.05 | 0.05 | 114 | 0.01 | 0.05 | 114 | -0.10 | 0.05 | Intervention | 0.31 | 0.581 |
|  | PLA | 114 | 0.12 | 0.09 | 114 | 0.00 | 0.05 | 114 | -0.05 | 0.05 | 113 | -0.08 | 0.05 | Inter*Time | 0.54 | 0.584 |
| Working Memory | MLE | 112 | 0.09 | 0.07 | 114 | -0.02 | 0.06 | 114 | -0.05 | 0.06 | 114 | -0.02 | 0.06 | Intervention | 0.13 | 0.724 |
|  | PLA | 114 | 0.07 | 0.06 | 114 | 0.05 | 0.06 | 114 | -0.10 | 0.06 | 113 | -0.01 | 0.06 | Inter*Time | 0.98 | 0.377 |
| Episodic Memory | MLE | 113 | 0.29 | 0.07 | 114 | -0.05 | 0.05 | 114 | -0.09 | 0.05 | 114 | -0.18 | 0.05 | Intervention | 0.54 | 0.466 |
|  | PLA | 114 | 0.26 | 0.07 | 114 | 0.05 | 0.05 | 114 | -0.08 | 0.05 | 114 | -0.20 | 0.05 | Inter*Time | 0.83 | 0.438 |

Inter = Intervention

# COMPASS Individual Tasks

**Table 2.** Performance on the COMPASS cognitive task individual outcomes. The Baseline data are raw means (plus SEM) collected prior to dosing. Data presented from the 30-, 180-, 300-minute post dose (p.d.) assessments are estimated means (plus SEM) derived from the linear mixed model analysis, with the final two columns showing test statistics (F) and associated probabilities (p) for each of the fixed effects included in the model.

|  |  |  | Baseline |  |  | 30 min p.d. | |  | 180 min p.d. | |  | 300 min p.d. | |  |  |  |
| --- | --- | --- | --- | --- | --- | --- | --- | --- | --- | --- | --- | --- | --- | --- | --- | --- |
|  |  | N | Mean | SEM | N | Mean | SEM | N | Mean | SEM | N | Mean | SEM |  | F | p |
| Simple Reaction Time (ms) | MLE | 113 | 361.36 | 10.26 | 114 | 391.02 | 13.92 | 114 | 378.77 | 13.92 | 114 | 375.71 | 13.92 | Intervention | 0.51 | 0.478 |
|  | PLA | 114 | 357.72 | 8.97 | 114 | 389.56 | 13.87 | 114 | 397.32 | 13.87 | 114 | 380.24 | 13.87 | Inter*Time | 0.35 | 0.703 |
| Choice Reaction Time % Accuracy | MLE | 113 | 96.30 | 0.32 | 114 | 96.31 | 0.29 | 114 | 96.12 | 0.29 | 114 | 95.67 | 0.29 | Intervention | 1.05 | 0.307 |
|  | PLA | 114 | 96.51 | 0.33 | 114 | 96.69 | 0.29 | 114 | 96.10 | 0.29 | 113 | 96.03 | 0.29 | Inter*Time | 0.56 | 0.574 |
| Choice Reaction Time (ms) | MLE | 113 | 444.83 | 5.77 | 114 | 474.26 | 6.82 | 114 | 453.70 | 6.82 | 114 | 460.04 | 6.82 | Intervention | 0.26 | 0.610 |
|  | PLA | 114 | 444.46 | 6.35 | 114 | 468.82 | 6.80 | 114 | 464.58 | 6.80 | 113 | 462.36 | 6.82 | Inter*Time | 1.35 | 0.260 |
| Digit Vigilance % Accuracy | MLE | 113 | 86.58 | 1.41 | 114 | 80.85 | 1.26 | 114 | 82.12 | 1.26 | 114 | 78.91 | 1.26 | Intervention | 0.11 | 0.740 |
|  | PLA | 114 | 87.33 | 1.13 | 114 | 80.32 | 1.25 | 114 | 81.14 | 1.25 | 114 | 79.74 | 1.25 | Inter*Time | 0.69 | 0.501 |
| Digit Vigilance RT (ms) | MLE | 113 | 467.74 | 3.48 | 114 | 485.12 | 2.43 | 114 | 480.24 | 2.43 | 114 | 483.78 | 2.43 | Intervention | 0.06 | 0.812 |
|  | PLA | 114 | 469.06 | 3.09 | 114 | 484.15 | 2.43 | 114 | 482.02 | 2.43 | 114 | 481.85 | 2.43 | Inter*Time | 0.70 | 0.496 |
| Digit Vigilance False alarms | MLE | 113 | 2.05 | 0.23 | 114 | 2.73 | 0.23 | 114 | 2.74 | 0.23 | 114 | 3.15 | 0.23 | Intervention | 0.22 | 0.644 |
|  | PLA | 114 | 1.99 | 0.20 | 114 | 2.87 | 0.23 | 114 | 2.88 | 0.23 | 114 | 3.09 | 0.23 | Inter*Time | 0.18 | 0.838 |
| Corsi Blocks Span score | MLE | 113 | 6.25 | 0.09 | 114 | 6.12 | 0.09 | 114 | 6.11 | 0.09 | 114 | 6.18 | 0.09 | Intervention | 0.36 | 0.551 |
|  | PLA | 114 | 6.23 | 0.09 | 114 | 6.29 | 0.09 | 114 | 6.05 | 0.09 | 114 | 6.19 | 0.09 | Inter*Time | 1.27 | 0.281 |

| Numeric Working Memory % Accuracy | MLE | 112 | 95.10 | 0.58 | 114 | 94.54 | 0.46 | 114 | 94.28 | 0.46 | 114 | 94.16 | 0.46 | Intervention | 0.01 | 0.906 |
| --- | --- | --- | --- | --- | --- | --- | --- | --- | --- | --- | --- | --- | --- | --- | --- | --- |
|  | PLA | 114 | 95.10 | 0.47 | 114 | 94.50 | 0.46 | 114 | 94.00 | 0.46 | 113 | 94.38 | 0.46 | Inter*Time | 0.29 | 0.751 |
| Numeric Working Memory RT (ms) | MLE | 112 | 736.98 | 15.20 | 114 | 741.03 | 10.13 | 114 | 730.35 | 10.13 | 114 | 717.27 | 10.12 | Intervention | 1.58 | 0.211 |
|  | PLA | 114 | 756.26 | 18.15 | 114 | 738.97 | 10.06 | 114 | 715.51 | 10.06 | 113 | 705.75 | 10.09 | Inter*Time | 0.43 | 0.651 |
| Peg and Ball Thinking time (ms) | MLE | 113 | 1745.68 | 78.31 | 114 | 1622.04 | 42.15 | 114 | 1584.64 | 42.14 | 114 | 1600.54 | 42.11 | Intervention | 0.01 | 0.946 |
|  | PLA | 114 | 1810.07 | 75.39 | 114 | 1621.20 | 41.98 | 114 | 1585.66 | 41.98 | 114 | 1593.31 | 41.98 | Inter*Time | 0.01 | 0.990 |
| Peg and Ball Completion time (ms) | MLE | 113 | 6397.52 | 134.44 | 114 | 6038.86 | 81.81 | 114 | 5912.78 | 81.81 | 114 | 5920.65 | 81.78 | Intervention | 0.01 | 0.946 |
|  | PLA | 114 | 6442.33 | 147.84 | 114 | 6083.39 | 81.52 | 114 | 5958.60 | 81.52 | 114 | 5842.33 | 81.52 | Inter*Time | 0.63 | 0.532 |
| Peg and Ball Errors (number) | MLE | 113 | 4.14 | 0.33 | 114 | 4.68 | 0.33 | 114 | 3.92 | 0.33 | 114 | 4.83 | 0.33 | Intervention | 0.23 | 0.635 |
|  | PLA | 114 | 4.45 | 0.34 | 114 | 4.33 | 0.33 | 114 | 4.27 | 0.33 | 114 | 4.47 | 0.33 | Inter*Time | 1.01 | 0.366 |
| Picture Recognition % Accuracy | MLE | 113 | 86.31 | 1.08 | 114 | 84.58 | 1.06 | 114 | 82.72 | 1.06 | 114 | 81.39 | 1.06 | Intervention | 1.19 | 0.277 |
|  | PLA | 114 | 86.73 | 1.06 | 114 | 82.85 | 1.06 | 114 | 82.65 | 1.06 | 114 | 81.04 | 1.06 | Inter*Time | 0.68 | 0.510 |
| Picture Recogniton RT (ms) | MLE | 113 | 820.93 | 14.36 | 114 | 816.32 | 11.73 | 114 | 809.97 | 11.73 | 114 | 786.42 | 11.73 | Intervention | 0.03 | 0.862 |
|  | PLA | 114 | 835.53 | 16.47 | 114 | 810.07 | 11.68 | 114 | 793.06 | 11.68 | 114 | 804.32 | 11.68 | Inter*Time | 1.40 | 0.248 |

| Word Recognition % Accuracy | MLE | 113 | 76.19 | 1.06 | 114 | 74.34 | 1.04 | 114 | 73.81 | 1.04 | 114 | 72.46 | 1.04 | Intervention | 1.15 | 0.286 |
| --- | --- | --- | --- | --- | --- | --- | --- | --- | --- | --- | --- | --- | --- | --- | --- | --- |
|  | PLA | 114 | 76.11 | 1.04 | 114 | 76.12 | 1.03 | 114 | 73.52 | 1.03 | 114 | 73.17 | 1.03 | Inter*Time | 0.89 | 0.411 |
| Word Recogniton RT (ms) | MLE | 113 | 879.50 | 22.60 | 114 | 855.89 | 16.18 | 114 | 852.58 | 16.18 | 114 | 832.60 | 16.18 | Intervention | 0.04 | 0.848 |
|  | PLA | 114 | 858.12 | 20.93 | 114 | 839.05 | 16.13 | 114 | 857.85 | 16.13 | 114 | 837.61 | 16.13 | Inter*Time | 0.49 | 0.614 |
| Immediate Word Recall % Accuracy | MLE | 113 | 47.85 | 1.44 | 114 | 44.38 | 1.15 | 114 | 44.33 | 1.14 | 114 | 45.47 | 1.14 | Intervention | 0.05 | 0.820 |
|  | PLA | 114 | 47.40 | 1.38 | 114 | 46.35 | 1.14 | 114 | 45.47 | 1.14 | 114 | 43.01 | 1.14 | Inter*Time | 2.63 | 0.073 |
| Delayed Word Recall % Accuracy | MLE | 113 | 31.68 | 1.51 | 114 | 18.19 | 1.28 | 114 | 18.14 | 1.28 | 114 | 14.36 | 1.28 | Intervention | 1.90 | 0.170 |
|  | PLA | 114 | 29.50 | 1.53 | 114 | 21.51 | 1.28 | 114 | 17.89 | 1.28 | 114 | 15.23 | 1.28 | Inter*Time | 1.51 | 0.221 |

Inter = Intervention

# Cognitive Demand Battery

**Table 3.** Performance on the Cognitive Demand Battery. The Baseline data are raw means (plus SEM) collected prior to dosing. Data presented from the 30-, 180-, 300-minute post dose (p.d.) assessments are estimated means (plus SEM) derived from the linear mixed model analysis, with the final two columns showing test statistics (F) and associated probabilities (p) for each of the fixed effects included in the model.

|  |  |  |  | Baseline | |  | 30 min p.d. | |  | 180 min p.d. | |  | 300 min p.d. | |  |  |  |
| --- | --- | --- | --- | --- | --- | --- | --- | --- | --- | --- | --- | --- | --- | --- | --- | --- | --- |
|  |  |  | N | Mean | SEM | N | Mean | SEM | N | Mean | SEM | N | Mean | SEM |  | F | p |
| Serial 3 subtractions Total (number) | Rep 1 | MLE | 113 | 46.14 | 1.64 | 114 | 48.76 | 1.20 | 114 | 48.89 | 1.20 | 114 | 49.60 | 1.20 | Intervention | 0.00 | 0.955 |
|  |  | PLA | 113 | 47.07 | 1.78 | 114 | 49.58 | 1.20 | 114 | 50.01 | 1.20 | 114 | 49.15 | 1.20 | Inter*Time | 0.53 | 0.588 |
|  | Rep 2 | MLE | 113 | 46.32 | 1.57 | 114 | 47.69 | 1.20 | 114 | 48.26 | 1.20 | 114 | 47.48 | 1.20 | Inter*Rep | 2.91 | 0.055 |
|  |  | PLA | 114 | 46.71 | 1.76 | 113 | 47.05 | 1.20 | 114 | 46.27 | 1.20 | 114 | 47.35 | 1.20 | Inter*Time*Rep | 0.61 | 0.766 |
|  | Rep 3 | MLE | 113 | 45.50 | 1.68 | 114 | 47.34 | 1.20 | 114 | 48.19 | 1.20 | 114 | 48.04 | 1.20 |  |  |  |
|  |  | PLA | 113 | 46.31 | 1.73 | 114 | 48.68 | 1.20 | 114 | 47.51 | 1.20 | 112 | 48.53 | 1.20 |  |  |  |
| Serial 3 subtractions Errors (number) | Rep 1 | MLE | 113 | 2.47 | 0.23 | 114 | 2.90 | 0.29 | 114 | 2.59 | 0.29 | 114 | 2.58 | 0.29 | Intervention | 7.78 | 0.005 |
|  |  | PLA | 113 | 1.95 | 0.22 | 114 | 2.80 | 0.29 | 114 | 2.32 | 0.29 | 114 | 2.51 | 0.29 | Inter*Time | 0.00 | 0.999 |
|  | Rep 2 | MLE | 113 | 2.32 | 0.21 | 114 | 2.56 | 0.29 | 114 | 2.83 | 0.29 | 114 | 2.92 | 0.29 | Inter*Rep | 1.92 | 0.147 |
|  |  | PLA | 114 | 2.20 | 0.23 | 113 | 2.65 | 0.29 | 114 | 2.39 | 0.29 | 114 | 2.40 | 0.29 | Inter*Time*Rep | 1.09 | 0.367 |
|  | Rep 3 | MLE | 113 | 2.39 | 0.22 | 114 | 3.59 | 0.29 | 113 | 2.67 | 0.30 | 114 | 3.13 | 0.29 |  |  |  |
|  |  | PLA | 113 | 2.65 | 0.25 | 114 | 2.51 | 0.29 | 114 | 2.26 | 0.29 | 112 | 2.63 | 0.30 |  |  |  |
| Serial 7 subtractions Total (number) | Rep 1 | MLE | 113 | 28.63 | 1.21 | 114 | 30.64 | 1.17 | 114 | 31.61 | 1.17 | 114 | 32.28 | 1.17 | Intervention | 0.66 | 0.417 |
|  |  | PLA | 114 | 28.61 | 1.37 | 113 | 32.15 | 1.17 | 114 | 32.32 | 1.17 | 114 | 32.32 | 1.17 | Inter*Time | 0.68 | 0.507 |
|  | Rep 2 | MLE | 113 | 29.50 | 1.27 | 114 | 30.64 | 1.17 | 114 | 30.88 | 1.17 | 113 | 31.86 | 1.17 | Inter*Rep | 2.77 | 0.063 |
|  |  | PLA | 114 | 29.97 | 1.26 | 114 | 30.03 | 1.17 | 114 | 31.11 | 1.17 | 114 | 31.30 | 1.17 | Inter*Time*Rep | 0.97 | 0.460 |
|  | Rep 3 | MLE | 113 | 29.64 | 1.31 | 114 | 30.11 | 1.17 | 114 | 32.21 | 1.17 | 113 | 31.89 | 1.17 |  |  |  |
|  |  | PLA | 114 | 30.80 | 1.38 | 114 | 31.38 | 1.17 | 114 | 31.84 | 1.17 | 111 | 32.00 | 1.17 |  |  |  |

| Serial 7 subtractions Errors (number) | Rep 1 | MLE | 113 | 2.50 | 0.49 | 114 | 3.16 | 0.28 | 114 | 2.81 | 0.28 | 114 | 2.97 | 0.28 | Intervention | 7.37 | 0.007 |
| --- | --- | --- | --- | --- | --- | --- | --- | --- | --- | --- | --- | --- | --- | --- | --- | --- | --- |
|  |  | PLA | 114 | 2.37 | 0.24 | 113 | 2.83 | 0.28 | 114 | 2.82 | 0.28 | 114 | 2.65 | 0.28 | Inter*Time | 1.46 | 0.233 |
|  | Rep 2 | MLE | 113 | 2.61 | 0.25 | 114 | 3.15 | 0.28 | 114 | 2.91 | 0.28 | 113 | 3.10 | 0.28 | Inter*Rep | 0.65 | 0.524 |
|  |  | PLA | 114 | 2.62 | 0.25 | 114 | 2.67 | 0.28 | 114 | 2.81 | 0.28 | 113 | 2.68 | 0.28 | Inter*Time*Rep | 0.21 | 0.990 |
|  | Rep 3 | MLE | 113 | 2.70 | 0.26 | 114 | 3.59 | 0.28 | 114 | 2.92 | 0.28 | 113 | 3.26 | 0.28 |  |  |  |
|  |  | PLA | 114 | 2.70 | 0.23 | 114 | 2.86 | 0.28 | 114 | 2.81 | 0.28 | 111 | 2.62 | 0.28 |  |  |  |
| RVIP Accuracy (%) | Rep 1 | MLE | 109 | 51.15 | 2.22 | 110 | 47.81 | 1.87 | 110 | 45.78 | 1.87 | 110 | 45.26 | 1.87 | Intervention | 0.75 | 0.388 |
|  |  | PLA | 111 | 50.83 | 2.04 | 111 | 46.36 | 1.87 | 111 | 48.82 | 1.87 | 111 | 45.01 | 1.87 | Inter*Time | 0.22 | 0.801 |
|  | Rep 2 | MLE | 109 | 46.90 | 2.23 | 110 | 43.38 | 1.87 | 109 | 44.60 | 1.87 | 110 | 41.16 | 1.87 | Inter*Rep | 0.09 | 0.913 |
|  |  | PLA | 111 | 47.23 | 2.13 | 111 | 44.91 | 1.87 | 111 | 44.26 | 1.87 | 111 | 41.71 | 1.87 | Inter*Time*Rep | 1.01 | 0.426 |
|  | Rep 3 | MLE | 108 | 45.28 | 2.22 | 110 | 44.05 | 1.87 | 109 | 43.83 | 1.87 | 109 | 40.59 | 1.87 |  |  |  |
|  |  | PLA | 111 | 45.43 | 2.15 | 111 | 43.75 | 1.87 | 111 | 43.95 | 1.87 | 110 | 41.54 | 1.87 |  |  |  |
| RVIP RT (ms) | Rep 1 | MLE | 109 | 529.28 | 5.76 | 110 | 525.58 | 5.64 | 110 | 516.08 | 5.64 | 110 | 526.68 | 5.64 | Intervention | 0.14 | 0.706 |
|  |  | PLA | 111 | 529.75 | 5.66 | 111 | 531.13 | 5.60 | 111 | 525.44 | 5.60 | 111 | 517.35 | 5.60 | Inter*Time | 1.53 | 0.217 |
|  | Rep 2 | MLE | 109 | 534.14 | 7.53 | 110 | 526.42 | 5.63 | 109 | 525.13 | 5.65 | 110 | 525.08 | 5.63 | Inter*Rep | 1.95 | 0.143 |
|  |  | PLA | 111 | 535.39 | 5.44 | 111 | 529.33 | 5.60 | 111 | 530.72 | 2.60 | 111 | 523.47 | 5.60 | Inter*Time*Rep | 0.47 | 0.876 |
|  | Rep 3 | MLE | 108 | 533.63 | 7.46 | 110 | 533.91 | 5.65 | 109 | 532.14 | 5.65 | 109 | 529.54 | 5.67 |  |  |  |
|  |  | PLA | 111 | 540.40 | 5.59 | 111 | 528.73 | 5.60 | 111 | 525.10 | 5.60 | 110 | 521.65 | 5.62 |  |  |  |

| RVIP False alarms (number) | Rep 1 | MLE | 109 | 3.50 | 0.48 | 110 | 3.91 | 0.47 | 110 | 4.40 | 0.47 | 110 | 4.07 | 0.47 | Intervention | 3.05 | 0.081 |
| --- | --- | --- | --- | --- | --- | --- | --- | --- | --- | --- | --- | --- | --- | --- | --- | --- | --- |
|  |  | PLA | 111 | 3.84 | 0.51 | 111 | 4.15 | 0.47 | 111 | 3.20 | 0.47 | 111 | 3.67 | 0.47 | Inter*Time | 0.60 | 0.550 |
|  | Rep 2 | MLE | 109 | 3.37 | 0.45 | 110 | 3.92 | 0.47 | 109 | 3.91 | 0.47 | 110 | 4.52 | 0.47 | Inter*Rep | 0.29 | 0.750 |
|  |  | PLA | 111 | 3.60 | 0.33 | 111 | 3.78 | 0.47 | 111 | 3.96 | 0.47 | 111 | 4.01 | 0.47 | Inter*Time*Rep | 0.87 | 0.539 |
|  | Rep 3 | MLE | 108 | 3.44 | 0.48 | 110 | 4.11 | 0.47 | 109 | 4.02 | 0.47 | 109 | 4.48 | 0.47 |  |  |  |
|  |  | PLA | 111 | 3.87 | 0.50 | 111 | 3.78 | 0.47 | 111 | 3.55 | 0.48 | 110 | 4.17 | 0.47 |  |  |  |
| Mental Fatigue | Rep 1 | MLE | 113 | 54.12 | 1.57 | 114 | 60.54 | 1.58 | 114 | 62.55 | 1.58 | 114 | 69.81 | 1.58 | Intervention | 1.04 | 0.309 |
|  |  | PLA | 114 | 51.42 | 1.64 | 114 | 58.84 | 1.59 | 114 | 63.28 | 1.59 | 114 | 68.85 | 1.59 | Inter*Time | 1.18 | 0.308 |
|  | Rep 2 | MLE | 113 | 60.11 | 1.70 | 114 | 64.55 | 1.58 | 113 | 67.45 | 1.58 | 114 | 73.28 | 1.58 | Inter*Rep | 0.05 | 0.953 |
|  |  | PLA | 114 | 58.30 | 1.69 | 114 | 63.02 | 1.58 | 114 | 67.56 | 1.58 | 114 | 71.95 | 1.58 | Inter*Time*Rep | 0.46 | 0.884 |
|  | Rep 3 | MLE | 113 | 65.55 | 1.74 | 114 | 66.88 | 1.59 | 114 | 68.69 | 1.59 | 113 | 73.22 | 1.59 |  |  |  |
|  |  | PLA | 114 | 63.08 | 1.92 | 114 | 63.66 | 1.58 | 114 | 68.68 | 1.58 | 113 | 73.59 | 1.58 |  |  |  |

Inter = Intervention; Rep = Repetition

# Mood and Profile of Mood States

**Table 4.** Data derived from scores on the Visual Analogue Mood Scales (VAMS), Stress Visual Analogue Scales (S-VAS) and the Profile of Mood States Questionnaire (POMS). The Baseline data are raw means (plus SEM) collected prior to dosing. Data presented from the 30-, 180-, 300-minute post dose (p.d.) assessments are estimated means (plus SEM) derived from the linear mixed model analysis, with the final two columns showing test statistics (F) and associated probabilities (p) for each of the fixed effects included in the model.

|  |  |  |  | Baseline | |  | 30 min p.d. | |  | 180 min p.d. | |  | 300 min p.d. | |  |  |  |
| --- | --- | --- | --- | --- | --- | --- | --- | --- | --- | --- | --- | --- | --- | --- | --- | --- | --- |
|  |  |  | N | Mean | SEM | N | Mean | SEM | N | Mean | SEM | N | Mean | SEM |  | F | p |
| VAMS | Alertness | MLE | 113 | 52.54 | 0.31 | 114 | 52.06 | 0.29 | 114 | 52.19 | 0.29 | 114 | 51.92 | 0.29 | Intervention | 3.19 | 0.076 |
|  |  | PLA | 114 | 52.23 | 0.31 | 114 | 52.75 | 0.29 | 114 | 52.35 | 0.29 | 114 | 52.14 | 0.29 | Inter*Time | 0.77 | 0.465 |
|  | Stress | MLE | 113 | 55.18 | 0.67 | 114 | 54.43 | 0.61 | 114 | 53.89 | 0.61 | 114 | 53.81 | 0.61 | Intervention | 0.02 | 0.886 |
|  |  | PLA | 114 | 55.78 | 0.69 | 114 | 54.13 | 0.61 | 114 | 54.54 | 0.61 | 114 | 53.27 | 0.61 | Inter*Time | 0.88 | 0.416 |
|  | Tranquility | MLE | 113 | 54.25 | 0.72 | 114 | 55.17 | 0.64 | 114 | 55.67 | 0.64 | 114 | 55.84 | 0.64 | Intervention | 0.58 | 0.447 |
|  |  | PLA | 114 | 54.20 | 0.72 | 114 | 55.55 | 0.64 | 114 | 56.29 | 0.64 | 114 | 55.84 | 0.64 | Inter*Time | 0.19 | 0.827 |
| S-VAS | Anxious | MLE | 113 | 41.31 | 1.69 | 114 | 42.42 | 1.50 | 114 | 44.58 | 1.50 | 114 | 46.08 | 1.50 | Intervention | 0.01 | 0.926 |
|  |  | PLA | 114 | 40.14 | 1.68 | 114 | 43.78 | 1.50 | 114 | 43.77 | 1.50 | 114 | 45.25 | 1.50 | Inter*Time | 0.65 | 0.522 |
|  | Stressed | MLE | 113 | 43.12 | 1.79 | 114 | 43.47 | 1.64 | 114 | 44.44 | 1.64 | 114 | 45.30 | 1.64 | Intervention | 0.08 | 0.779 |
|  |  | PLA | 114 | 42.49 | 1.81 | 114 | 43.18 | 1.64 | 114 | 43.86 | 1.64 | 114 | 45.28 | 1.64 | Inter*Time | 0.03 | 0.969 |
|  | Relaxed | MLE | 113 | 51.33 | 1.41 | 114 | 54.19 | 1.54 | 114 | 52.35 | 1.54 | 114 | 50.44 | 1.54 | Intervention | 0.25 | 0.618 |
|  |  | PLA | 114 | 52.03 | 1.65 | 114 | 52.33 | 1.53 | 114 | 51.24 | 1.53 | 114 | 51.78 | 1.53 | Inter*Time | 0.86 | 0.426 |
|  | Calm | MLE | 113 | 55.82 | 1.42 | 114 | 54.55 | 1.60 | 114 | 57.31 | 1.61 | 114 | 53.84 | 1.60 | Intervention | 0.55 | 0.460 |
|  |  | PLA | 114 | 55.92 | 1.53 | 114 | 54.46 | 1.59 | 114 | 56.90 | 1.59 | 114 | 52.00 | 1.59 | Inter*Time | 0.23 | 0.795 |

| POMS | Anger-Hostility | MLE | 108 | 41.37 | 0.50 | 113 | 41.23 | 0.36 | 111 | 41.45 | 0.36 | 112 | 41.10 | 0.36 | Intervention | 0.54 | 0.464 |
| --- | --- | --- | --- | --- | --- | --- | --- | --- | --- | --- | --- | --- | --- | --- | --- | --- | --- |
|  |  | PLA | 110 | 41.13 | 0.50 | 113 | 41.13 | 0.36 | 113 | 41.51 | 0.36 | 113 | 41.76 | 0.36 | Inter*Time | 1.24 | 0.292 |
|  | Confusion-Bewilderment | MLE | 108 | 42.82 | 0.80 | 113 | 42.64 | 0.48 | 111 | 42.41 | 0.48 | 112 | 42.90 | 0.48 | Intervention | 2.73 | 0.100 |
|  |  | PLA | 110 | 42.23 | 0.79 | 113 | 43.12 | 0.47 | 113 | 43.16 | 0.47 | 113 | 43.36 | 0.47 | Inter*Time | 0.16 | 0.850 |
|  | Depression-Dejection | MLE | 109 | 43.95 | 0.75 | 113 | 44.05 | 0.36 | 111 | 43.86 | 0.36 | 112 | 43.68 | 0.36 | Intervention | 0.72 | 0.398 |
|  |  | PLA | 110 | 44.05 | 0.75 | 113 | 44.19 | 0.36 | 113 | 44.03 | 0.36 | 113 | 44.22 | 0.36 | Inter*Time | 0.31 | 0.731 |
|  | Fatigue-Inertia | MLE | 109 | 40.22 | 0.77 | 113 | 41.49 | 0.60 | 111 | 41.11 | 0.60 | 112 | 42.45 | 0.60 | Intervention | 0.86 | 0.354 |
|  |  | PLA | 110 | 40.28 | 0.76 | 113 | 41.60 | 0.59 | 113 | 41.31 | 0.59 | 113 | 43.30 | 0.59 | Inter*Time | 0.40 | 0.669 |
|  | Tension-Anxiety | MLE | 109 | 39.27 | 0.75 | 113 | 39.51 | 0.48 | 111 | 39.42 | 0.48 | 112 | 39.76 | 0.48 | Intervention | 0.29 | 0.589 |
|  |  | PLA | 110 | 39.85 | 0.80 | 113 | 39.75 | 0.48 | 113 | 39.02 | 0.48 | 113 | 39.36 | 0.48 | Inter*Time | 0.62 | 0.540 |
|  | Vigor-Activity | MLE | 109 | 46.38 | 0.85 | 113 | 44.85 | 0.63 | 110 | 44.12 | 0.64 | 111 | 42.19 | 0.63 | Intervention | 2.98 | 0.086 |
|  |  | PLA | 110 | 46.08 | 0.94 | 113 | 44.71 | 0.63 | 113 | 45.42 | 0.63 | 113 | 43.41 | 0.63 | Inter*Time | 1.58 | 0.208 |
|  | Total Mood Disturbance | MLE | 107 | 44.72 | 0.92 | 113 | 45.33 | 0.52 | 110 | 45.35 | 0.52 | 111 | 46.09 | 0.51 | Intervention | 0.08 | 0.781 |
|  |  | PLA | 110 | 44.72 | 0.92 | 113 | 45.54 | 0.51 | 113 | 45.21 | 0.51 | 113 | 46.38 | 0.51 | Inter*Time | 0.33 | 0.716 |
|  | Friendliness | MLE | 109 | 47.05 | 0.93 | 113 | 45.34 | 0.68 | 111 | 45.28 | 0.68 | 112 | 42.60 | 0.68 | Intervention | 0.11 | 0.737 |
|  |  | PLA | 110 | 47.07 | 0.98 | 113 | 44.82 | 0.68 | 113 | 44.80 | 0.68 | 113 | 43.14 | 0.68 | Inter*Time | 0.82 | 0.439 |

Inter = Intervention
